# Case Report: Complete Remission of a Patient With Metastatic Gastric Cancer Treated With Nivolumab Combined With Chemotherapy After Palliative Surgery

**DOI:** 10.3389/fimmu.2022.908558

**Published:** 2022-06-29

**Authors:** Peilin Dai, Xi Rao, Xi Zhang, Enming Qiu, Gang Wu, Yu Lin, Sitong Li, Zhou Li, Zhai Cai, Shuai Han

**Affiliations:** ^1^ The Second School of Clinical Medicine, Southern Medical University, Guangzhou, China; ^2^ Department of Gastrointestinal Surgery, General Surgery Center, Zhujiang Hospital, Southern Medical University, Guangzhou, China; ^3^ Department of Oncology, Zhujiang Hospital, Southern Medical University, Guangzhou, China; ^4^ Department of Pathology, Zhujiang Hospital, Southern Medical University, Guangzhou, China; ^5^ Burning Rock Biotech, Guangzhou, China

**Keywords:** immune checkpoint inhibitor, nivolumab, advanced gastric cancer, complete remission, case report

## Abstract

Metastatic advanced gastric cancer, for which treatment strategies are extremely limited, has a poor prognosis. Complete remission is rare. Patients usually lose the opportunity of therapeutic surgery because the lesions cannot be completely removed, although it can greatly prolong their survival time. Palliative surgery usually suggests bad outcomes. In recent years, the immune checkpoint inhibitor (ICI) nivolumab has shown significant efficacy in the treatment of advanced gastric cancer. However, its applicable conditions and optimal withdrawal time remain controversial owing to its low response rate and high incidence of immune-related adverse events. Herein, we introduce a 66-year-old male patient with advanced gastric cancer with multiple liver metastases who underwent laparoscopic total gastrectomy for acute gastric bleeding. The patient received eight cycles of S-1 plus oxaliplatin (SOX) and switched to eight cycles of SOX plus nivolumab combined regimen in a stable state, later achieving complete remission. There was no recurrence for 32 months after the surgery. This is the first reported case of gastric cancer with multiple liver metastases with long-term complete remission with nivolumab treatment after palliative surgery. The potential mechanism of complete remission was discussed through clinical, genomic, and immune characteristics. The patient had a history of psoriasis and was positive for programmed death ligand 1 (PD-L1), and the interaction of *TP53* mutation and *HER-2* (-) gene may be associated with complete remission.

## Introduction

Metastatic advanced gastric cancer (AGC) has a poor prognosis, and its treatment is extremely limited. The reported 5-year survival rates range from 8.8% to 14.9% (1–3), and complete remission (CR) is rare. Nevertheless, conversion surgery can improve the prognosis of patients with unresectable gastric cancer (4, 5), but only a few patients with multiple metastases undergo surgery to alleviate symptoms, and it is usually limited to palliative resection or bypass surgery (6, 7). Patients usually lose the opportunity of therapeutic surgery to prolong survival because the lesion cannot be completely removed. Palliative surgery usually suggests a disappointing outcome (8). Furthermore, nivolumab/opdivo is a human immunoglobulin (Ig)G4 monoclonal antibody against programmed cell death protein 1 (PD-1) (9). PD-L1 binds to PD-1 on cytotoxic T lymphocytes (CTLs) to increase the level of immunosuppressive factors in the tumor microenvironment or impede the activation and transport of CTLs to tumors to help the tumor escape immune monitoring. Blocking the PD-1/PD-L1 axis by anti-PD-1 antibody (aPD-1) or anti-PD1 ligand 1 (aPD-L1) can reactivate CTLs in tumors and enhance the anti-tumor effect of various advanced solid tumors, such as lung cancer, melanoma, and gastric cancer (10–14). In recent years, nivolumab has shown significant survival benefits in the treatment of AGC, which has been confirmed by a phase 3 clinical trial (ATTRACTION-2) (15). It is generally believed that PD-L1-positive gastric cancer with a high tumor mutation burden (TMB-H) and high microsatellite instability (MSI-H) can obtain a good response. However, although the case we discuss here was PD-L1-positive and had medium TMB and microsatellite stability (MSS), there was still an encouraging prognosis. Analyzing the differences is useful to clarify the mechanism of good response. Herein, we report a successful clinical case of chemotherapy combined with immunotherapy after laparoscopic gastrectomy for AGC. Our case provides a novel treatment idea: for the patients with metastatic gastric cancer sensitive to anti-PD-1 therapy, palliative surgery followed by anti-PD-1 combined chemotherapy is a potential scheme to achieve a better prognosis than non-surgical treatment.

The significant anti-tumor effect, low response rate, and high incidence of immune-related adverse events (IRAEs) of nivolumab make its applicable conditions and optimal withdrawal time controversial (16–18). The ATTRACTION-2 trial has shown that the complete response rate was only 1.1% in the overall population, and 50% of the patients exhibited early disease progression. A study revealed that when nivolumab was administered to AGC patients, Eastern Cooperative Oncology Group Performance Status (ECOG PS) of >1, liver metastasis, and large tumor size at baseline were significantly associated with hyperprogression (HPD) (19). Our case had the above high-risk predictors, but the overall survival (OS) was 32 months, which was significantly superior to the median OS (5.26–6.77) (15, 20) of metastatic gastric cancer. Therefore, it is uncertain whether there are some joint effects at the clinical, genomic, and immune levels to improve anti-tumor response. Hence, studying its applicable conditions from multiple levels is extremely necessary. Additionally, for those who get CR, the vast majority choose continuous medication to prevent recurrence. When to stop giving nivolumab and how long the CR state can be maintained after the withdrawal unless intolerable IRAEs emerge are yet to be known. Our patient did not experience recurrence after complete withdrawal.

IRAEs represent the *off-target effect* caused by nivolumab, which coexists with the *on-target effect*, i.e., the anti-tumor effect. A proportion of patients can achieve long-term survival through immunotherapy, and patients with IRAEs have a longer survival time than those without (18, 21). Our patient had an excellent response to nivolumab. The clinical, immune, and genomic characteristics of patients have great reference significance for the research related to AGC treatment. Different from the previously reported cases of successful use of nivolumab after chemotherapy failure, this case completely cleared metastasis with anti-PD-1 in a stable state.

## Case Description

### Demographic Information and Clinical Findings

A 66-year-old retired policeman was hospitalized in July 2019 due to upper abdominal filling, swallowing disorder, and significant weight loss for 3 months. Previously, he had suffered from chronic gastritis and taken omeprazole irregularly for 4 years, the curative effect was not significant. There was no other pertinent comorbidity except psoriasis. There was no tobacco or alcohol dependence, no history of tumor, a similar clinical manifestation, and no familial genetic disease. Physical examination showed epigastric tenderness without abnormalities of other organ systems. The height of the patient was 150 cm and his weight was 60 kg.

### Diagnostic Assessments

**Figure 1:**
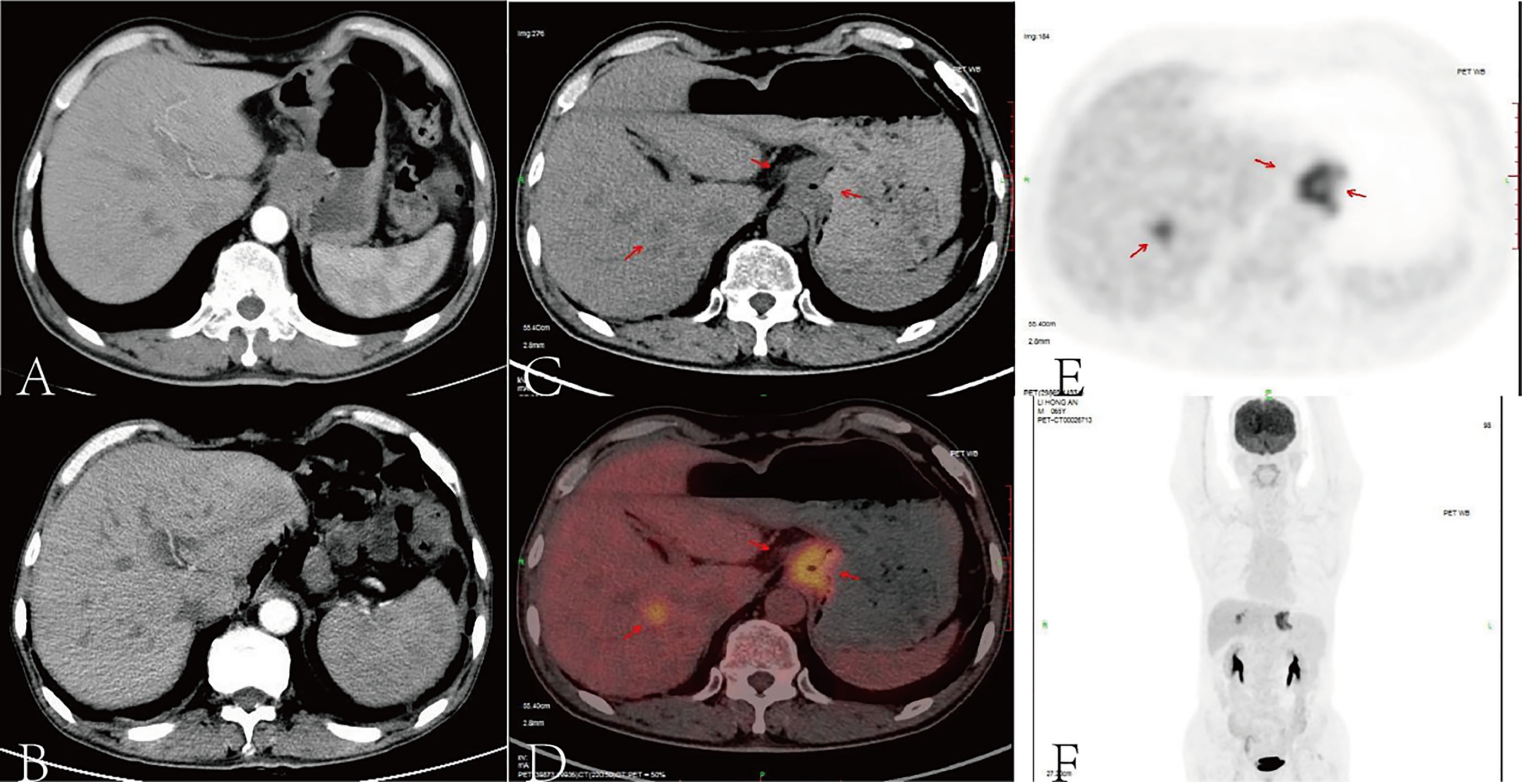
According to an abdominal contrast-enhanced computed tomography (CT) scan, there were a malignant gastric mass and seven low-density shadows in the liver recognized as liver metastases **(A)**. After eight cycles of SOX, contrast-enhanced CT scan showed no recurrence of the primary tumor, but liver metastases did not show any obvious changes **(B)**. After surgery and chemotherapy for gastric cancer, the stomach was absent, and the lower esophagus and jejunum were anastomosed. A high-density metal anastomotic line shadow (indicated by the arrow) can be seen at the anastomotic stoma, and a small patch of slightly increased radiation uptake shadow can be seen locally. A small nodular low-density shadow with a size of about 0.5 cm can be seen in the right lobe of the liver (S8 segment). The density and radioactivity distribution in other parts of the liver were not abnormal. A small nodule-calcified shadow can be seen under the capsule of the right lobe of the liver (S6 segment). According to the positron emission tomography (PET) scan, the red arrows pointed out the tumor foci **(C–E)**, the image **(F)** showed the image of coronal plane.

Gastroscopy showed a mass in the gastric fundus extending to the cardia of the stomach. The pathological examination (**Supplementary Figure 1**) of the specimen proved to be poorly differentiated adenocarcinoma with *Helicobacter pylori* (+). According to an abdominal contrast-enhanced computed tomography (CT) scan, there were a malignant gastric mass and seven low-density shadows in the liver recognized as liver metastases (**Figure 1A**). Additionally, the patient tested positive for PD-L1 (tumor proportion score [TPS] = 3%, immune proportion score [IPS] = 10%). With a tumor mutational burden [TMB] of 8.06 Muts/Mb and an ECOG PS of 1, the tumor was categorized as moderate TMB. However, acute gastric hemorrhage, a serious complication of AGC, occurred in this patient. After multidisciplinary treatment according to the patient’s opinion, we performed laparoscopic total gastrectomy with regional lymph node dissection and esophagojejunostomy. During the surgery, a mass of about 6 × 5 × 4 cm was found in the gastric fundus, which had invaded the serosa. Multiple liver metastases were unresectable during the surgery. The pathological examination of intraoperative tissue specimen showed that the tumor had invaded laminae muscularis mucosae and nerves. Concurrently, the patient was diagnosed with pT4aN0M1 stage IV GC. Immunohistochemistry (IHC) demonstrated the following (**Supplementary Figure 2**): CK (+), CK7 (+), *HER-2* (–), Ki67 approximately 95% (+), CD45 (-), SYN (-), and *EBER* (-). The next-generation sequencing (NGS) of circulating tumor DNA analysis (3D Medicines Co., Shanghai) revealed two gene mutations: *TP53* p.T125T (39.22%) and *FLCN* p.S113Afs*17 (21.66%), with no mutation in epidermal growth factor receptor (*EGFR*) (-), *ERBB2/HER2* (-), and *KRAS* (-). The tumor microenvironment (TME) of the patient was microsatellite stability (MSS: MLH1/MSH2/MSH6/PMS2).

### Therapeutic Interventions and Follow-Up

We treated the patient with S-1 (40 mg twice a day on days 1–14) plus oxaliplatin (130 mg/m^2^ once on day 1) (SOX) as first-line chemotherapy for eight cycles (3 weeks per cycle) due to his liver metastases. Subsequently, a contrast-enhanced CT scan demonstrated that there was no recurrence in the primary tumor, but the liver metastases did not show any obvious changes (**Figure 1B**). Thus, we planned to treat the patient with SOX plus nivolumab (200 mg on day 1 once every 2 weeks) due to the patient’s strong desire to clear the metastasis and its satisfactory ECOG PS and PD-L1 expression. After four cycles of nivolumab, an enhanced CT scan showed that the liver lesions were significantly reduced. The effectiveness of immunotherapy led us to decide to maintain the treatment regimen.

**Figure 2:**
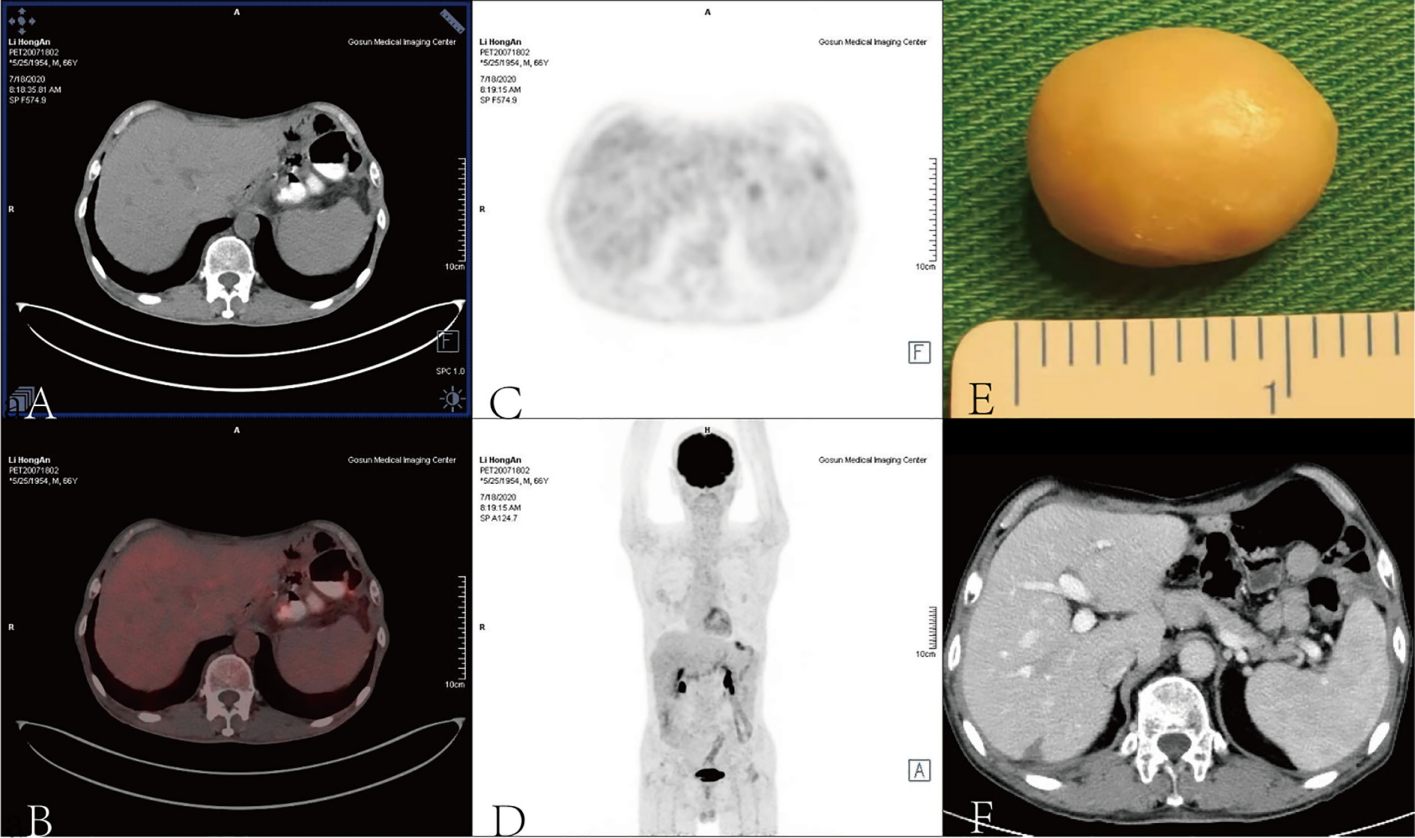
** **A surgical intervention was implemented to remove the calcified liver metastasis **(E)** when pneumonia resolved, and PET **(A–D)** and CT scans **(F)** demonstrated that liver metastasis disappeared.

**Figure 3:**
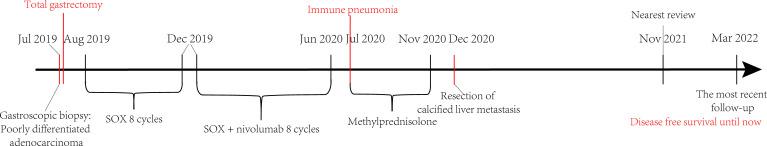
The course timeline showed that shortly after the diagnosis of advanced gastric cancer, the patient underwent total gastrectomy for acute gastric bleeding, followed by eight cycles of SOX and then eight cycles of SOX + nivolumab treatment. Subsequently, immune pneumonia occurred and was treated with methylprednisolone. After the patient became stable, the suspected calcified metastasis of the liver was removed, and all medications were stopped. The patient has achieved disease-free survival until now.

A positron emission tomography (PET) scan was performed after the eighth cycle of SOX and showed a resectable liver metastasis and no recurrence of the primary tumor in the stomach (**Figures 1C–F**). However, the patient suffered from cough, asthma, and dyspnea. A CT scan showed inflammation and fibroproliferative foci in the right lung and the upper lingual segment and the posterior basal segment of the lower lobe of the left lung, and there were multiple small nodules scattered in both lungs and bilateral pleural thickening. Considering immune pneumonia (grade III), nivolumab was discontinued, methylprednisolone (1 mg/kg daily) was applied for treatment, and the subsequent CT scan showed fewer inflammatory lesions than before. Concurrently, we had to use SOX alone instead of combining it with nivolumab. The patient received a total of 16 cycles of SOX, and the cumulative dose of oxaliplatin was 1,040 mg/m^2^. Dose-related adverse reactions, such as peripheral neurotoxic symptoms, were not observed. Additionally, the patient—considering his history of psoriasis for 25 years—observed significantly more severe dermatitis with itching during nivolumab treatment. Thereafter, surgical intervention was implemented to remove the resectable liver metastasis after recovery from pneumonia. The pathology confirmed that there were extensive lymphocytes aggregated without tumor cells in the resected tissue, and then PET and CT scan showed that liver metastasis disappeared (**Figures 2A–F**). The multidisciplinary discussion considered that all examinations had confirmed that the patient’s primary and secondary lesions had been completely cleared; thus, all anti-tumor treatments were stopped. The timeline and the dynamic changes in tumor markers are displayed in **Figure 3** and **Supplementary Figure 2**. The patient was discharged home on a postoperative day and discontinued any drugs. He achieved a complete response and remained relapse-free for 32 months, with no evidence of disease (NED) recurrence in the stomach and liver.

## Discussion

The REGATTA trial revealed that resection of GC with a single incurable factor may lead to a worse outcome (8). Nevertheless, this case is contrary to that conclusion. Similar to the results of the latest CheckMate 577 clinical trial (22), our case demonstrated that adjuvant chemotherapy with nivolumab can improve the prognosis of patients undergoing gastrectomy (23). However, the clinical indications and prognostic significance of surgery after treatment with nivolumab remain obscure (24). Some studies have shown that for AGC patients with distant metastasis, only gastrectomy and lymph node dissection were performed, and then CR was observed after chemotherapy (25). Moreover, the median survival time of the operation group was significantly longer than that of the non-operation group (4). Whether the palliative surgery of the primary focus provides benefits for the later immune checkpoint blocker to exert its anti-tumor effect is a question worthy of exploration. It might shorten the course of immune checkpoint inhibitor (ICI) treatment, reduce the scope of tumors, and increase the concentration of drugs in a small range of tumors to avoid critical IREAs emerging before the satisfactory response. It might also provide a longer time window for weighing the optimal withdrawal time. Emerging evidence has indicated that angiogenesis and immunosuppression frequently occur simultaneously (26); accordingly, the resection of primary lesions may inhibit angiogenesis and enhance anti-tumor immunity.

Liver metastasis is usually considered a predictor of poor response to nivolumab (27). During the first four cycles of SOX treatment, there was no recurrence of gastric anastomosis, and liver metastases had neither significant progress nor regression, indicating that tumor cells did not respond well to SOX. The limitation of the presented case is the absence of molecular and immunological characterization of the secondary lesions. There are three hypotheses:

(1) The primary tumor was sensitive to SOX; thus, it did not relapse. Liver metastases were not sensitive to SOX but sensitive to nivolumab since these were heterogenous from primary tumors. Some studies have shown that gastroesophageal cancer is a heterogeneous disease, and there is heterogeneity within and between tumors (28–30). Different from gastroesophageal cancer, our case displays gastric cardia cancer, although their anatomical position is close. Because there are no clinical data on the contrast between primary tumors and metastatic ones, it is unknown whether it is heterogeneous from the primary tumor, but the possibility still exists.
(2) Nivolumab overcomes the resistance of the tumor to SOX or strengthens the effect of SOX on tumor cells. Concurrently, nivolumab also plays an anti-tumor role. Studies have shown that nivolumab exposure may promote the subsequent chemosensitivity of patients with AGC (3, 31, 32). Moreover, nivolumab monotherapy has been reported to cause serious IRAEs, resulting in the failure of nivolumab restart in tumor recurrence. The combination of extended immunity and targeted therapy does not aggravate the adverse reactions of patients (33). Therefore, we propose another hypothesis: the combination of drugs can reduce the probability of adverse events caused by nivolumab or delay their occurrence.
(3) After the resection of the primary tumor, the tumor cells were no longer active, had no response to SOX at all, and only showed a good response to nivolumab. A 3-year follow-up study has confirmed the long-term efficacy of nivolumab monotherapy (34). Nonetheless, previous studies have shown that there is little difference between the efficacy of SOX plus nivolumab and nivolumab alone. The mechanism needs to be elucidated by further clinical research.

**Figure 4:**
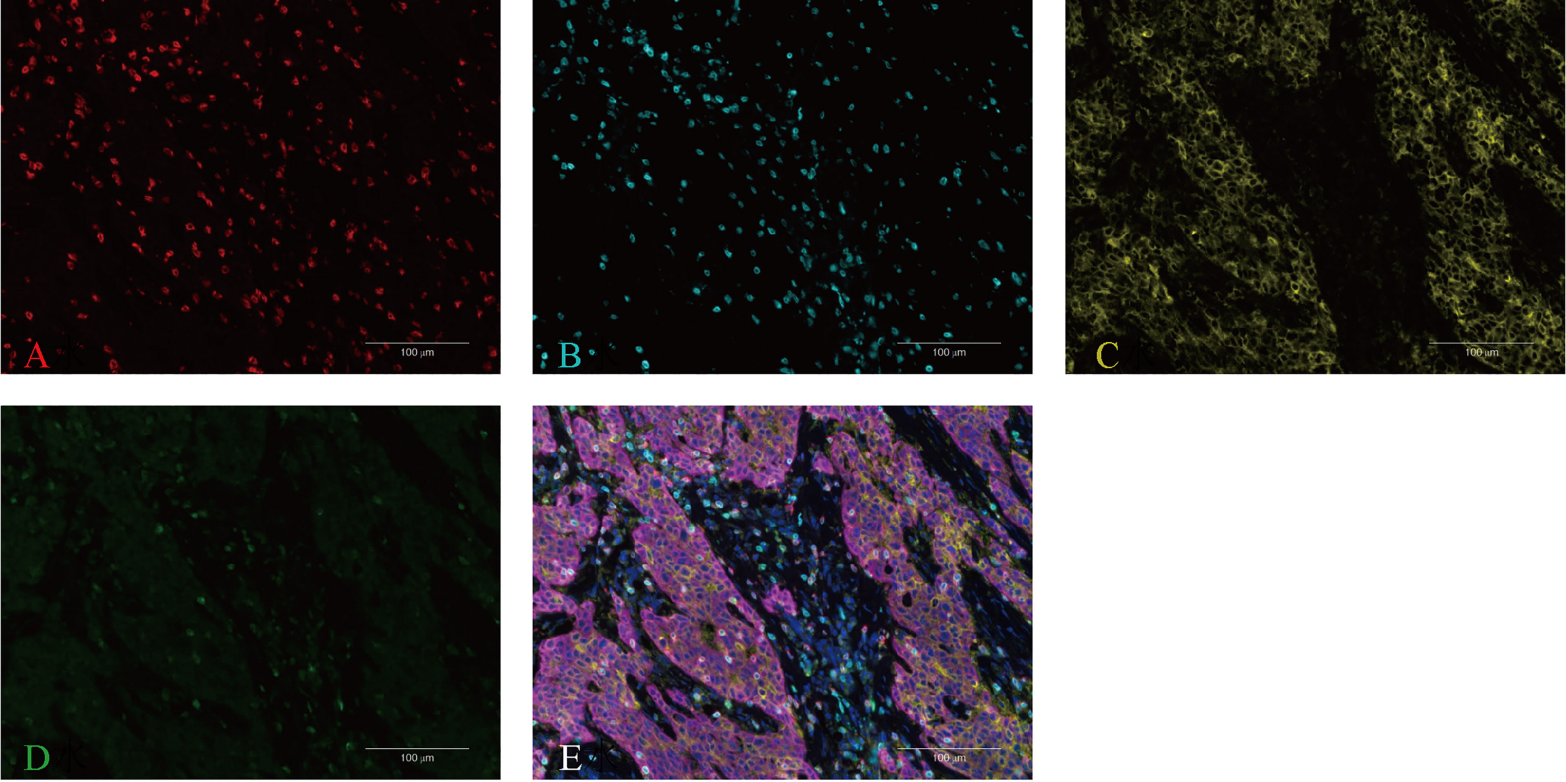
Multiplex immunofluorescence staining for CD8 **(A)**, CD3 **(B)**, PD-L1 **(C)**, PD-1 **(D)**, and the merged image **(E)** in the primary tumor tissue. The multiplex IHC platform, which can detect the expression of multiple markers in a single section, was utilized to measure the expression of PD- 1/PD-L1 and immune cell infiltration. The immune markers included CD8+ TIL and CD3+ TIL.

The effectiveness of our regimen has also been confirmed by the results of subsequent CheckMate 649 and KEYNOTE-590 studies: first-line chemotherapy combined with nivolumab in the treatment of PD-L1-positive patients may provide survival benefits (35, 36). Notwithstanding, other studies illustrated that nivolumab can be applied in the first-line treatment of AGC regardless of the expression of PD-L1 (37). In the ATTRACTION-4 trial, the efficacy of nivolumab combined with S-1/capecitabine + oxaliplatin in the treatment of untreated GC has been encouraging (38). In this case, the tumor microsatellite has been stable, a different finding from most previous studies. Additionally, it has been demonstrated that microsatellite status had no significant effect on median progression-free survival (PFS) (35, 39, 40). The ECOG PS 1 in our case confirmed that the prognosis of patients with a PS of ≤1 is better than in those with a PS of ≥2 (40). In contrast, it has been reported that for GC PD-L1-negative patients with high-efficiency mismatch repair (PMMR)/MSS and TMB-1, immune checkpoint blockade can also achieve good outcomes after systematic treatment failure (41–43). One article reported that a patient received only four doses of nivolumab, discontinued any anti-tumor treatment in the next 12 months, and successfully achieved complete remission and long-term maintenance. Resembling our case, the characteristics of the tumor presented tumor microsatellite stability and that the mutation load was 6.35 Muts/Mb (medium) in blood. Moreover, the anti-tumor effect still existed 2 years after the cessation of nivolumab (44). It has been reported that (45) due to the fear of recurrence, the persistent application of nivolumab remained in the third year after CR, and long-term medication would bring a great economic burden and high risk of adverse immune reactions to patients. Therefore, more studies are required to evaluate the optimal withdrawal time. Additionally, our patient was characterized by a history of active psoriasis for about 25 years. However, in the landmark CheckMate 649 clinical trial, patients with a known or suspected history of autoimmune diseases have been excluded. Consequently, our patient did not technically belong to the group represented by the study, but the curative effect was superior to that of this group, indicating that the history of psoriasis might bring a potentially positive effect on anti-tumor response. On biopsy, the abundant lymphocytes in the hepatic resected tissue also proved that cellular immunity played an important role in killing tumor cells. It may be of value to study the molecular mechanism affecting the reactivity of ICIs from the perspective of autoimmune-related genes or the molecular mechanism of an immune response. In view of the immune evaluation in the nivolumab phase 1 study, lymphocytes play a critical part in drug action (46), and the pathogenesis of psoriasis is also closely associated with lymphocyte immune imbalance. The tumor immune microenvironment could reflect the immune response, several studies have reported that PD-L1 expression and tumor infiltrating lymphocytes (TILs) were associated with better response to immunotherapy (47–51). Thus, we performed a multiplex immunohistochemistry assay (Microenvironment Analysis Panel, Burning Rock Biotech, Guangzhou, China) to measure the density of CD3+/CD8+ TILs, PD-L1 and PD-1 in the primary tumor tissue (**Supplementary Table 1**). Our results suggest that, CD3+/CD8+ TILs were positive in cancer nests and stroma. In addition, PD-L1 expression was positive and mainly located in cancer nests (**Figure 4**). These findings indicate that the patient presented with an “PD-L1+/TIL+” microenvironment, which might be associated with the underlying mechanism for disease remission in immunotherapy.

Although the differentiated histological type is an advantage of AGC (52), this case was a poorly differentiated adenocarcinoma with a good response to nivolumab. Distinct from our case, previous studies have suggested that *HER-2*-positive status is related to the improvement of prognosis (18, 53). Furthermore, some studies have proposed that the co-expression of *HER-2* and PD-L1 may contribute to the immune escape of GC, meaning that *HER-2*-negative status creates favorable conditions for effective immunotherapy (12). Compared with *EGFR* wild-type patients, patients with advanced non-small cell lung cancer with *EGFR* mutation show poor efficacy of PD-1/PD-L1 inhibitor treatment (54). In this case, *FLCN* p.s113afs * 17 mutation caused *FLCN* protein to lose the DENN domain, which reduces EGFR protein activity mediated by epidermal growth factor (EGF) by activating Rab35, thereby inhibiting the *EGFR* signal pathway (55, 56). It is likely that the final effect of *EGFR* mutation differs in the inhibition of the *EGFR* signaling pathway induced by *FLCN*, and *FLCN* mutation is more conducive to the anti-tumor effect of nivolumab. Compared with *KRAS* wild-type patients, patients with *KRAS* mutation benefit more from PD-1 inhibitor treatment (57, 58). Another mutation site of our patient was located at the last position of exon-4 of *TP53* gene, which can affect the normal shear of mRNA influencing the arrest function of p53 protein in the cell cycle, and weakening the ability of induced apoptosis (59). Patients with non-small cell lung cancer with *TP53* or *KRAS* mutation are more sensitive to PD-1/PD-L1-related therapy, especially those with co-mutation (55). Additionally, patients with *TP53* mutation have a higher CD8-T cell density than those with *STK11* or *EGFR* mutation; hence, they respond better to ICIs (60). NGS in this case showed *TP53* and *FLCN* mutations, with no mutation in *EGFR* (-), *HER-2* (-), and *KRAS* (-). Based on the above-described analysis, *TP53* mutation, *HER-2* (-), and *EGFR* (-) are the favorable conditions for a good response to nivolumab, while *KRAS* wild type is the unfavorable factor. Obviously, the advantages of this genome type in anti-PD-1 treatment outweigh the disadvantages, and the prognosis is good.

## Conclusion

Whether molecular biomarkers can accurately predict the outcome of immunotherapy remains controversial. However, it is speculated that whether the arrangement and combination of multiple biomarkers can improve the prediction accuracy is worth exploring. In conclusion, we reported a case of stage IV GC that successfully achieved CR through palliative surgery, chemotherapy, and subsequent immunotherapy. Nonetheless, supported by only one single case, our conclusion should be proved by accumulating more cases and summarizing the efficacy of a similar regime and the case characteristics of the successful use of nivolumab to achieve CR and then contribute to the establishment of more accurate comprehensive treatment strategies.

## Data Availability Statement

The original contributions presented in the study are included in the article/**Supplementary Material**. Further inquiries can be directed to the corresponding author.

## Ethics Statement

The studies involving human participants were reviewed and approved by the Ethics Committee of Zhujiang Hospital. The patients/participants provided their written informed consent to participate in this study.

## Author Contributions

PD, XR, and XZ researched data and wrote the manuscript. PD, EQ, GW, and YL analyzed the inspection data and contributed to the discussion. SL, ZL, ZC, and SH reviewed and modified the manuscript. All authors contributed to the article and approved the submitted version.

## Funding

This work was supported by Special Funds for the Cultivation of Guangdong College Students’ Scientific and Technological Innovation (Grant No. pdjh2021a0094). The foundation plays a role in encouraging college students to take an active part in scientific and technological innovation. The authors declare that all sources of funding received for the research being submitted and the funds are received for open access publication fees from the above grant.

## Conflict of Interest

Author SL was employed by company Burning Rock Biotech.

The remaining authors declare that the research was conducted in the absence of any commercial or financial relationships that could be construed as a potential conflict of interest.

## Publisher’s Note

All claims expressed in this article are solely those of the authors and do not necessarily represent those of their affiliated organizations, or those of the publisher, the editors and the reviewers. Any product that may be evaluated in this article, or claim that may be made by its manufacturer, is not guaranteed or endorsed by the publisher.
