# Supplementary material for: Case Report: Complete Remission of a Patient With Metastatic Gastric Cancer Treated With Nivolumab Combined With Chemotherapy After Palliative Surgery
Source: Front Immunol. 2022 Jun 29;13:908558. doi: 10.3389/fimmu.2022.908558 (PMC9278084; doi:10.3389/fimmu.2022.908558)
Supplement: Supplementary file 1 [file DataSheet_1.docx]

Supplementary Material

# Supplementary Figures

**
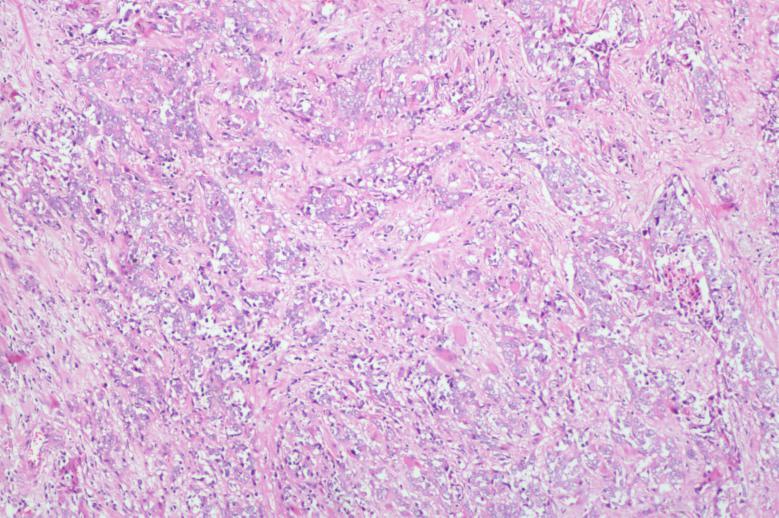
**

**Supplementary Figure 1.** The pathological examination (×100) of the specimen proved to be poorly differentiated adenocarcinoma. Tumor cell positive proportional fraction (TPS): TPS = number of PD-L1 membrane staining positive tumor cells of any intensity / total number of tumor cells * 100%. The staining morphology of cell membrane is discontinuous linear, circular or basolateral staining, which is acceptable. The staining intensity of cell membrane is acceptable after excluding the interference of background color, i.e. ≥ 1 +.


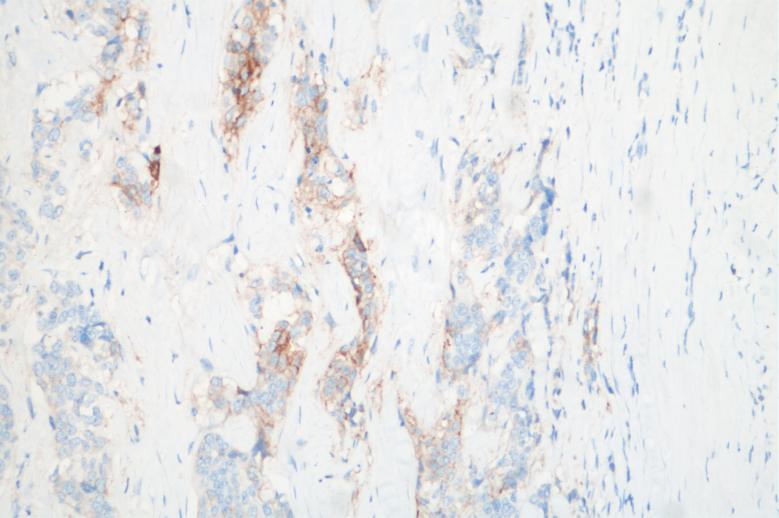


**Supplementary Figure 2.** The immunohistochemistry (IHC×200) demonstrated: CK (+), CK7 (+), HER-2 (-), Ki67 approximately 95% (+), CD45 (-), SYN (-) and EBER (-). Tumor immune cell positive proportional fraction (IPS): IPS = number of tumor related immune cells with PD-L1 membrane staining and cytoplasmic positive at any intensity / total number of tumor related immune cells * 100%. the staining morphology of cell membrane is discontinuous linear, circular or basolateral staining, which is acceptable. The staining intensity of cell membrane is acceptable after excluding the interference of background color, i.e. ≥ 1 +.


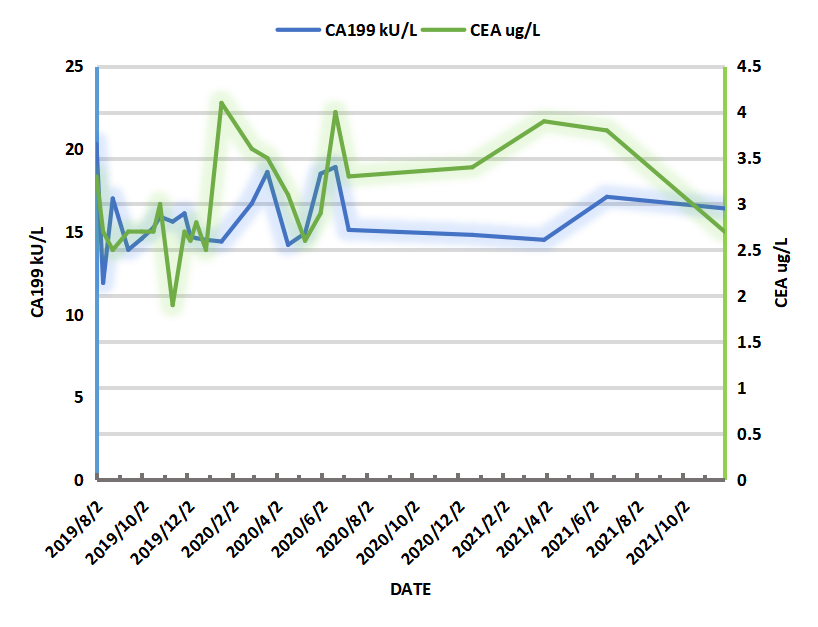


**Supplementary Figure 3.** The dynamic changes of tumor markers (CA199 and CEA). The marker levels fluctuated greatly in the early stage, and the level of tumor markers was stable in the later stage.

# Supplementary Table

**Supplementary Table 1.** Multiplex immunohistochemical predictive biomarkers.

| **Biomarker** | **Density (No./mm^2^)** | **Positive rate (%)** |
| --- | --- | --- |
| Tumor CD8 | 369 | 3.79 |
| Stroma CD8 | 502 | 7.36 |
| Tumor CD3 | 322 | 3.31 |
| Stroma CD3 | 917 | 13.43 |
| Tumor PD-L1 | 1733 | 17.78 |
| Stroma PD-L1 | 107 | 1.57 |
| Tumor PD-1 | 23 | 0.24 |
| Stroma PD-1 | 53 | 0.78 |
